# The prognostic role of p53 and its correlation with CDK9 in urothelial carcinoma

**DOI:** 10.1007/s12094-022-02994-6

**Published:** 2022-11-14

**Authors:** Jędrzej Borowczak, Krzysztof Szczerbowski, Mateusz Maniewski, Marek Zdrenka, Piotr Słupski, Hanna Andrusewicz, Joanna Łysik-Miśkurka, Paula Rutkiewicz, Magdalena Bodnar, Łukasz Szylberg

**Affiliations:** 1 Department of Obstetrics, Gynaecology and Oncology, Chair of Pathomorphology and Clinical Placentology, Collegium Medicum in Bydgoszcz, Nicolaus Copernicus University in Torun, University Hospital No. 2 in Bydgoszcz, Ujejskiego 75, 85-168 Bydgoszcz, Poland; 2 Department of Tumor Pathology and Pathomorphology, Oncology Centre-Prof. Franciszek Łukaszczyk Memorial Hospital, Bydgoszcz, Poland; 3 Department of Urology, University Hospital No. 2 im. Dr. Jan Biziel in Bydgoszcz, Bydgoszcz, Poland; 4 Chair of Pathology, University Hospital No. 2 im. Dr. Jan Biziel in Bydgoszcz, Bydgoszcz, Poland

**Keywords:** P53, CDK9, Bladder cancer, Expression, Prognosis

## Abstract

**Purpose:**

The mutation of p53 is considered a pivotal step in bladder cancer pathogenesis. Recently, distinct interactions between p53 and CDK9, a transcription regulator, have been described. In this work, we explored the prognostic role of p53 expression and evaluated its associations with CDK9 in urothelial carcinoma.

**Materials and methods:**

The research group consisted of 67 bladder cancer samples and 32 normal urothelial mucosa samples. All specimens were analyzed using ImageJ and the IHC profiler plugin. To validate the results, 406 cases from The Cancer Genome Atlas database were analyzed.

**Results:**

P53 and CDK9 are overexpressed in urothelial cancer tissues when compared to normal urothelial tissues (*p* < 0.05). High p53 expression was observed in metastatic tumors and tumors with high CDK9 expression (*p* < 0,05). High p53 expression was predictive for shorter survival in patients with non-muscle-invasive bladder cancer (HR = 0.107 [0.012–0.96]; *p* = 0.046) but did not correlate with prognosis in the muscle-invasive group. In high CDK9 cancers, high p53 expression correlated with the occurrence of high-grade and muscle-invasive tumors (*p* < 0.05).

**Conclusion:**

High expression of p53 correlates with unfavorable clinical features of bladder cancer. CDK9 is associated with the expression of p53, possibly through interactions with p53 inhibitors. Since the blockade of CDK9 in other malignancies reactivates wild-p53 activity, confirming the crosstalk between p53 and CDK9 in bladder cancer may be another step to explain the mechanism of tumor progression in its early stages.

## Introduction

Bladder cancer is one of the most common malignancies worldwide, with approximately 524,000 cases annually [1]. In 2019, its incidence and mortality rates increased and were estimated at 6.5 and 2.9 per 100 000, respectively, accounting for 229,000 deaths and 4.39 million disability-adjusted life years [1]. In many European countries, the prevalence of bladder cancer is still on a rise, presumably due to the popularity of smoking and an aging population [2]. The survival time and rate depend on early diagnosis; the 5-year survival reaches up to 95.8% among those with an in situ disease, 69.5% in localized disease, and only 4.6% in metastatic cancer [2]. Although 51% of all patients are diagnosed with carcinoma in situ, others are usually not suitable for radical treatment. Urothelial carcinoma (BLCA) is the most common histologic type of bladder cancer and constitutes approximately 90% of all cases [3]. The genetic abnormalities that accumulate during the progression of the disease may prevent the apoptosis of cancer cells and hinder the efficacy of systemic therapy [4]. In such cases, genomic profiling may be a key point to truly personalize care for bladder cancer patients. Therefore, finding new prognostic markers and therapeutic targets seems of great importance.

Tumor protein 53 (p53) is a major tumor suppressor encoded by the *TP53* gene located on human chromosome 17 [5]. P53 is post-translationally stabilized and activated in response to cellular stress, including DNA damage, hypoxia, and mitogenic oncogenes [6]. By intervening in the activity of its direct target genes, such as cyclin-dependent kinases, DNA repair genes, or apoptotic proteins, p53 alleviates cellular stress, maintains genome integrity, and prevents the initiation of carcinogenesis [5, 6]. TP53 is mutated in about half of human cancers. The hereditary loss of p53 function is associated with the occurrence of aggressive cancers, especially in young patients [7]. Therefore, p53 has become a potential therapeutic target. Recently, its newly discovered interactions with cyclin-dependent kinases have shed new light on how its activity may influence the early steps of tumorigenesis [8].

Cyclin-dependent kinases are a family of kinases that must bind to their regulatory proteins, cyclins, to gain enzymatic activity [9]. Cyclin-dependent kinase 9 (CDK9) is a transcription-regulating protein that has recently gained attention after promising in-vivo and in-vitro trials in multiple human cancers [10]. CDK9 binds to cyclin T, forming positive transcription elongation factor-B (P-TEFb), and stimulates transcription through the activity of RNA polymerase II (RNA POL II). Its overexpression may cause the accumulation of anti-apoptotic proteins, such as MYC or Mcl-1, disrupt cellular homeostasis, and promote the immortalization of abnormal cells [8, 10]. As a central regulatory hub of transcription, CDK9 is required for cell proliferation, differentiation, and apoptosis. It is also believed to partake in tumor growth via the p53-related pathway [11, 12]. Currently, two isoforms are known: 42-kDa and 55-kDa; they may differ functionally and prognostically. The upregulation of CDK9 42-kDa was recently associated with increased cell proliferation and survival, while no such activity of CDK9 55-kDa was detected [13, 14]. Instead, the 55-kDa isoform seems to mediate DNA repair through the Ku70-associated pathway, suggesting its potential role in maintaining genomic stability [15].

Recently, two novel drug regimens, immune checkpoint inhibitors, and fibroblast growth factor receptor tyrosine kinase inhibitors, have been approved for the treatment of bladder cancer. Nevertheless, frequent chemoresistance and low response rates prompt further research for novel therapeutic targets. In this work, we evaluate the prognostic value of p53 in urothelial carcinoma and investigate its possible correlations with CDK9 expression.

## Materials and methods

### Patients and tissue samples

The study included paraffin-embedded blocks containing tissue samples that were collected from urothelial carcinoma patients treated in the Department of Urology. The research group consisted of 67 bladder cancer samples, while 32 normal urothelial mucosa samples were used as a control group. All samples were collected during either transurethral resection of bladder tumor (TURBT) or radical cystectomy (RC). Clinical data, including age, sex, tumor grade and stage, cancer invasiveness, lymph node metastases, tumor size, the occurrence of progression and recurrence, as well as overall survival time were obtained (Table 1). The study was conducted following the Declaration of Helsinki, and the protocol was approved by the Bioethics Committee (KB881/2019).

**Table 1:** Clinicopathological characteristics of the study group

Variables	*n* (%)
Age (mean)	73.37 years (range 45–88 years)
Median follow-up time	60 months (range 5–60 months)
Sex	
Female	11/67 (16.42%)
Male	56/67 (83.58%)
Grade	
Low (G1)	32/67 (47.76%)
High (G2. G3)	35/67 (52.24%)
Stage	
Ta-T1	36/67 (53.73%)
T2	19/67 (28.36%)
T3	8/67 (11.94%)
T4	4/67 (0.06%)
Lymph node invasion	
N0	56/67 (83.58%)
N1-3	9/67 (13.43%)
Unknown	2/67 (0.03%)
Distant metastasis	
No	57/67 (85.07%)
Yes	7/67 (10.45%)
Unknown	3/67 (4.48%)
Size	
< 3 cm	31/67 (46.27%)
≥ 3 cm	36/67 (53.73%)
Invasiveness	
NMIBC	33/67 (49.25%)
MIBC	33/67 (49.25%)
Unknown	1/67 (1.5%)
Type of procedure	
TURBT	33/67 (49.2%)
RC	29/67 (43.28%)
Unknown	5/67 (7.46%)
Progression	
No	32/67 (47.76%)
Yes	16/67 (23.88%)
Unknown	19/67 (28.36%)
Recurrence	
No	7/67 (10.48%)
Yes	23/67 (24.33%)
Unknown	37/67 (55.22%)
Mean recurrence time	13.0 months (range 0–60 months)
Disease course	
Alive	40/67 (59.7%)
Dead	27/67 (40.3%)

### Sample staining

A retrospective immunohistochemical analysis of p53 comprised 67 formalin-fixed, paraffin-embedded tissue blocks derived from 67 bladder cancer patients. The tissue block was cut into 5 μm sections, attached to a glass slide, and incubated at 60 °C for 2 h. IHC staining was performed on the Ventana Benchmark Ultra platform according to NordiQC operating procedure. A primary p53 monoclonal antibody (Bp53-11) was used for staining.

The expression of CDK9 was determined using IHC assays according to the protocol described in Buchholz et al. study [16]. In the beginning, standardization and optimization of the IHC method were performed on a recommended tissue, based on the antibody datasheet and reference sources (The Human Protein Atlas: https://www.proteinatlas.org; [17]). In brief, 3 μm thick sections of the tissue arrays were baked for 1 h at 60 °C before xylene deparaffinization and subsequent rehydration through graded ethanol (99.8, 96, 90 and 80%). Tissue sections were incubated with a primary rabbit monoclonal anti-CDK9 antibody (1:200, 40 min; ab76320, Abcam). Primary antibodies were visualized using either the UltraView Universal DAB Detection Kit (Roche Diagnostics/Ventana) followed by color development using 3,3-diaminobenzidine. The slides were counterstained with Hematoxylin II for 12 min and Bluing Reagent for 4 min. Finally, tissue sections were dehydrated in increasing ethanol concentrations (80, 90, 96, and 99.8%), cleared in xylenes (I–IV), mounted using a mounting medium, and examined.

### Image acquisition and immunohistochemical analysis

The immunohistochemically stained slides were scanned by Ventana DP 200 Slide scanner (Roche Diagnostics). For each sample, two experienced pathologists selected the most representative regions and captured images at x10 magnification with a VENTANA Image Viewer v. 3.2.0. The analysis was performed using the ImageJ 1.53j version (NIH, Bethesda, Maryland) (Java 1.8.0_172) and the IHC profiler plugin. The expressions of p53 and CDK9 were assessed by following the standard protocol designed by Varghese et al. [18]. The highly positive zone was found to be ranging from 1 to 60; 61 to 120 for the positive zone; 121 to 170 for the low positive zone; and 181 to 220 for the negative zone, respectively. The intensity values ranging from 221 to 255 predominantly represent fatty tissues, stroma, or background artifacts that do not contribute to pathological scoring and were therefore excluded from the score determination zones. For each sample, the expression of p53 and CDK9 was obtained by calculating the H-Score. H-score was assigned using the formula [1 × (% cells low positive) + 2 × (% cells positive) + 3 × (%cells high positive)], obtaining a value from 0 to 300.

### In silico analysis

The analysis was carried out using the data gathered from The Human Pathology Atlas (www.proteinatlas.org), cBioPortal [19] and The Cancer Genome Atlas (TCGA) database [20]. The TCGA cohort consisted of 406 patients diagnosed with urothelial carcinoma. The TCGA RNA-seq data were mapped using the Ensembl gene id available from TCGA, and the FPKMs (Fragments Per Kilobase of exon per Million reads) for TP53 and CDK9 were used to perform the quantitative analysis of their expression. The patients were classified into two expression groups based on the FPKM value. The best cutoffs were chosen using the Cutoff Finder web app [21]. Cancers with an expression of TP53 lower than 23.5 FPKM were considered low-TP53 and those with an expression equal to or higher than 23.5 FPKM were classified as high-TP53. Similarly, if the expression of CDK9 was lower than 13, the tumors were classified as low CDK9, otherwise were considered high-CDK9.

### Statistical analysis

All statistical analyses were performed using Statistica version 13.3 (Statsoft) and Microsoft Excel 2019. The *p* value was considered statistically significant if *p* < 0.05. Continuous variables were tested for normality by the Kolmogorov–Smirnov test. The relations between groups of categorical variables were analyzed in the Mann–Whitney *U* Test or the ANOVA Kruskal–Wallis test. Correlations between clinicopathological features and p53 expression were evaluated using Pearson’s correlation coefficient or Spearman’s rank correlation coefficient. Univariate and multivariate analyses of potential predictors of overall survival were performed using Cox proportional hazard regression. Results were expressed as hazard ratio (HR) and 95% confidence interval (CI). The two-sided *p* value of < 0.05 was considered to indicate statistical significance. The relation between p53 expression with overall survival was evaluated with a log-rank test and presented using the Kaplan–Meier estimate.

## Results

### Patients characteristics

**Fig. 1:**
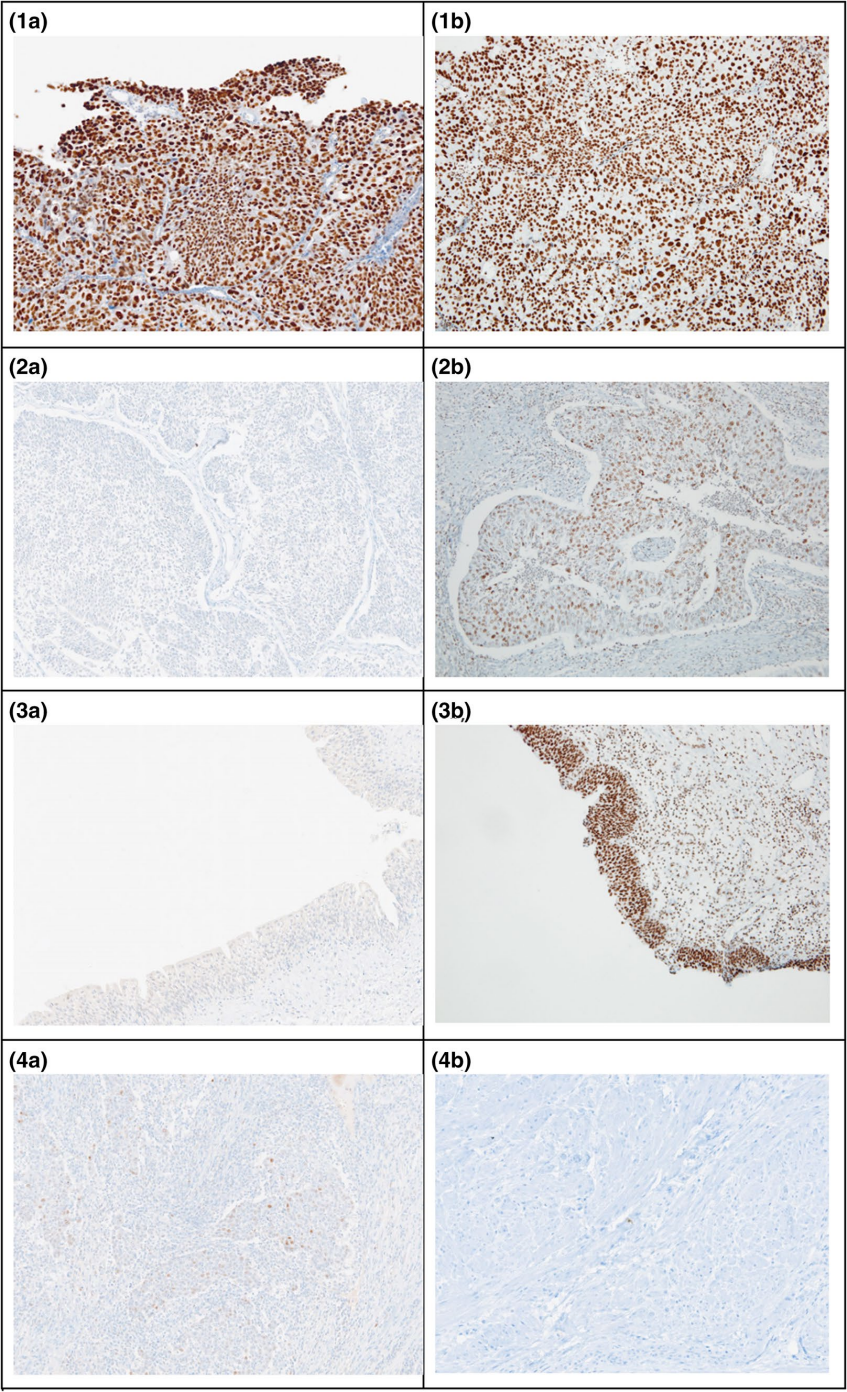
Representative cross-sectional staining patterns of **1a** bladder cancer with high p53 expression; **1b** bladder cancer with high CDK9 expression; **2a** bladder cancer with low p53 expression; **2b** bladder cancer with low CDK9 expression; **3a** normal mucosa with low p53 expression; **3b** normal mucosa with high CDK9 expression and positive reaction in the cells of the stromal inflammatory infiltration; **4a** p53 negative control; **4b** CDK9 negative control

The research group consisted of 11 female and 56 male patients; their mean age was 71.5 years (range 45–88 years) and the median follow-up time was 5 years. Among 67 patients, 32 (47.76%) were diagnosed with low-grade tumors and 35 (52.24%) were diagnosed with high-grade tumors. 36 (53.73%) tumors were low-stage (Ta/T1), while 31 were high-stage (46.27%; T2–T4). At the time of diagnosis 9 (13.43%) patients had lymph node metastases and 7 (9.72%) had distant organ metastases. The mean 5-year overall survival time was 45.26 months, ranging from 5.0 to 60.0 months. The characteristics of this cohort are summarized in Table 1 (Fig. 1).

### P53 is overexpressed in urothelial carcinoma

**Fig. 2:**
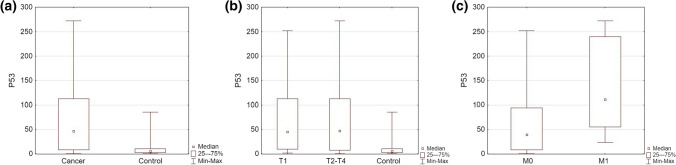
P53 expression: **a** cancer vs. control (*p* = 0.00001), **b** T1 and T2–T4 vs. control (*p* = 0.0001), **c** in non-metastatic cancers vs. cancers with distant metastasis (M0 vs M1; *p* = 0.02)

Immunohistochemical staining was evaluated in all samples in the study and the control group. The immunoreactivity observed in bladder cancer samples was significantly higher than in the control group (median H-SCORE = 46 vs. 5, respectively; *p* = 0.00001), and the results retained significance in both high-stage and low-stage tumors (Fig. 2a, b). The expression of p53 was then classified into low and high p53 expression groups with the cutoff being set at 90 H-Score.

### P53 expression and clinical features of urothelial carcinoma

We evaluated the correlations between p53 expression and clinical features of BLCA. P53 levels were significantly higher in tumors with distant metastases than in non-metastatic tumors (*p* = 0.02) (Fig. 2c). There were no differences in p53 expression between groups of various stages, grades, invasiveness, tumor size, and lymph node invasion (*p* > 0.05) (Table 2).

**Table 2:** Correlations between p53 expression and clinicopathological features

Clinical data	Total *N*	Median P53 expression (min–max)	Q1	Q3	Statistical differences between groups (*p* < 0.05)
Cancer group	67	46 (0–272)	8	112	*p* = 0.00001
Control group	26	5 (0–85)	2	10	
Low-grade	32	47.5 (1–252)	11	95.5	*p* = 0.84
High-grade	35	40 (0–272)	7	129	
T1	36	45 (1–252)	9	112	*p* = 0.88
T2–T4	31	47 (0–272)	7	112	
NMIBC	33	49 (1–210)	11	113	*p* = 0.51
MIBC	33	39 (0–272)	7	73	
N0	56	46.5 (0–272)	9	113.5	*p* = 0.89
N1–N3	9	55 (0–252)	23	63	
M0	57	39 (0–252)	8	94	*p* = 0.021
M1	7	111 (23–272)	55	240	
Progression	16	70.5 (7–272)	31	187	*p* = 0.19
No progression	32	45 (1–252)	11	113.5	
Low CDK9	49	39 (0–272)	7	80	*p* = 0.027
High CDK9	18	77 (5–252)	13	207	

*NMIBC* non-muscle-invasive bladder cancer; *MIBC* muscle-invasive bladder cancer;* N0, N1-N3* lymph nodes metastasis, *M0, M1* distant metastasis, *Q* quartile

The prognostic value of p53 was evaluated separately in muscle-invasive bladder cancer (MIBC) and non-muscle-invasive bladder cancer (NMIBC) patients. In the NMIBC group, patients with high p53 expression had significantly lower overall survival rate (94.44 vs. 57.14%, respectively; *p* = 0.015), lower progression-free survival rate (91.74 vs 52.85%, *p* = 0.013) and higher risk of reduced disease-free survival (HR = 9.63 [1.06–87.67); *p* = 0.04) than patients with low p53 expression after 5 years of follow-up. Univariate analysis revealed that low p53 expression (HR = 0.107 [0.012–0.96]; *p* = 0.046), low tumor grade (HR = 0.15 (0.03 − 0.093), *p* = 0.04) and a lack of distant metastases (HR = 0.06 [0.01–0.37]; *p* = 0.002) were favorable prognostic factors for longer patients’ survival in NMIBC (Table 3). P53 was not prognostic of patients' survival in the MIBC group.

**Table 3:** Univariate analysis of patients’ overall survival in non-muscle-invasive bladder cancer

Variable	HR	95% CI	*p* value
Age (< 70 vs. > 70)	0.53	0.06–4.79	0.57
Sex (F vs. M)	0	0–0	0.995
Stage (T1 vs. T2–T4)	0.14	0.015–1.27	0.08
Grade (low vs. high)	0.15	0.03-0.093	0.04
Metastasis (M0 vs. M1)	0.06	0.01–0.37	0.002
Tumor size (< 3 cm vs. > 3 cm)	0.37	0.06–2.2	0.27
Recurrence (Y/N)	0.52	0.05–5.73	0.59
CDK9 (low vs. high)	1.05	0.18–6.29	0.96
P53 (low vs. high)	0.11	0.01–0.96	0.046

### CDK9 is overexpressed in bladder cancer and correlates with longer survival

CDK9 staining intensity was measured in normal tissue and bladder cancer samples. CDK9 was overexpressed in the cancer group when compared to the control (196 vs. 166 H-Score, respectively). The expression of CDK9 was also higher in low-grade, non-muscle-invasive, and lower-stage tumors compared to high-grade, muscle-invasive, and high-stage tumors, respectively (*p* < 0.05). The samples were then classified into high-CDK9 and low-CDK9 expression groups, with the cutoff point being 219 H-Score. Patients with high CDK9 expression had a significantly higher 5-year survival rate than patients with low CDK9 tumors (76.19 vs. 51.93%; *p* = 0.04).

### Correlations between the expression of p53 and CDK9

**Fig. 3:**
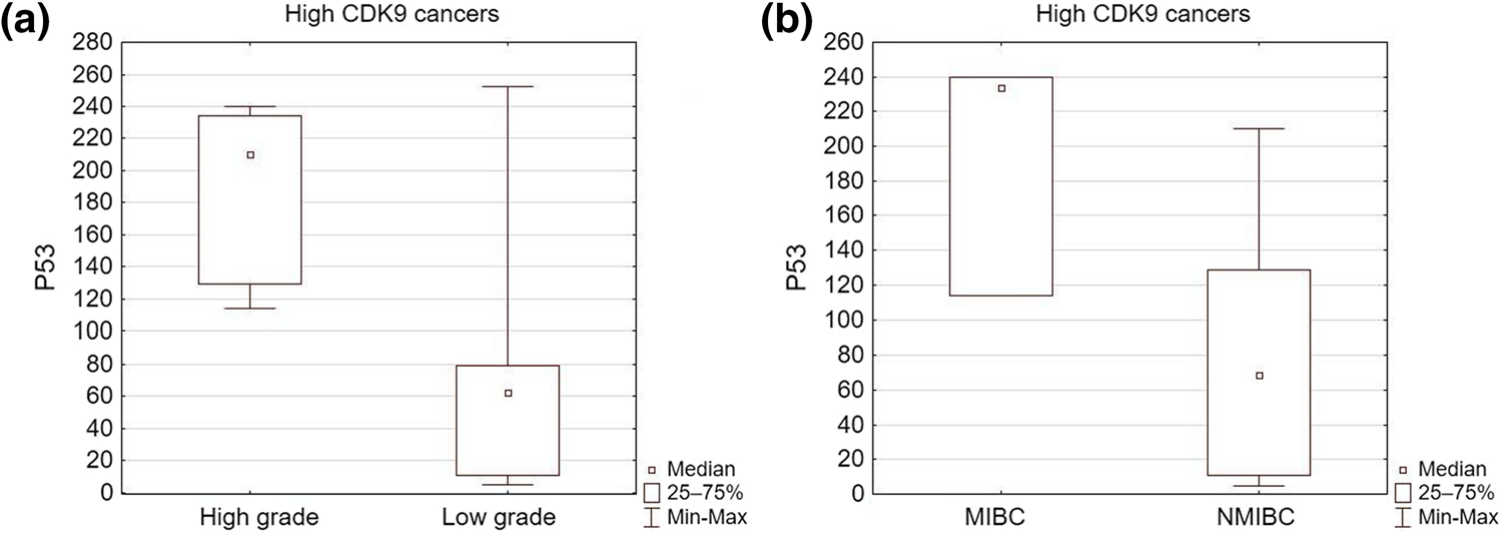
P53 expression in high CDK9 urothelial cancers depending on: **a** tumor grade (*p* = 0.02), **b** tumor invasiveness (*p* = 0.037) *MIBC* muscle-invasive bladder cancer, *NMIBC* non-muscle-invasive bladder cancer

We examined correlations between the expression of p53 and CDK9. Tumors with high CDK9 expression showed significantly higher p53 expression than those with low CDK9 (mean H-SCORE 79.5 vs 39, respectively; *p* < 0.05) (Table 2); however, no significant correlation between p53 and CDK9 expressions in the research group was found (Pearson’s correlation coefficient *k* = 0.14; *p* > 0.05). In tumors with high CDK9, higher p53 expression was detected in high-grade and muscle-invasive cancers compared to low-grade and non-muscle-invasive tumors (*p* < 0.05) (Fig. 3).

### In silico analysis of CDK9 and p53 correlations in urothelial carcinoma

**Fig. 4:**
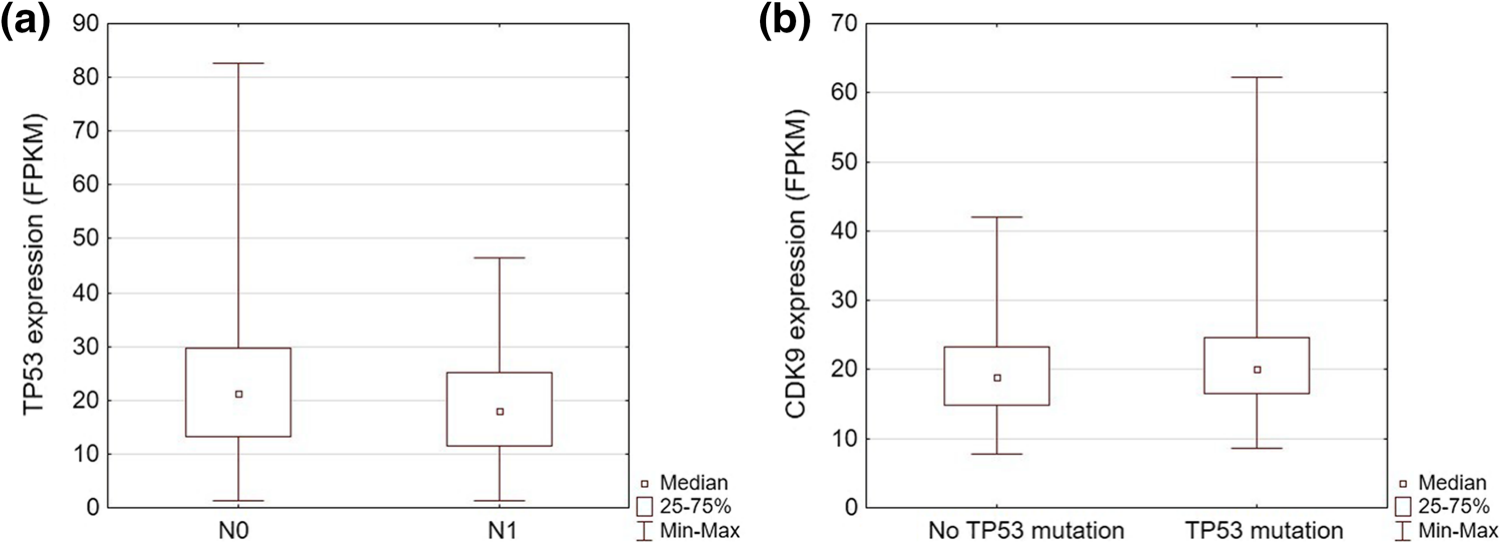
Statistically significant results of the TCGA cohort analysis: **a** TP53 expression in the TCGA cohort depending on the status of lymph node invasion (*p* = 0.012), **b** CDK9 expression depending on the presence of TP53 mutation (*p* = 0.012)

To validate our findings, in silico analysis of p53 and CDK9 expression was performed. We accessed The Human Pathology Atlas (www.proteinatlas.org) and gathered the corresponding data from The Cancer Genome Atlas (TCGA) database [31]. The TCGA cohort consisted of 406 patients diagnosed with urothelial carcinoma. The basic patient characteristics are summarized in Table 4. The median age of patients was 69 years (range 34–90 years) and the median follow-up was 1.46 years (Table 4). The patients were dichotomized into low and high-expression groups. In the TCGA cohort, high CDK9 expression correlated with longer overall survival and favorable clinical features of urothelial carcinoma [22]. We found that tumors with no lymph node metastasis showed higher TP53 levels than those with lymph node metastasis (median FPKM 21.35 vs 18.00; *p* < 0.05) (Fig. 4a). The expression of TP53 was not prognostic of patients’ survival in this group and did not differ between tumors of different stages, grades, or distant metastatic status (*p* > 0.05). There was also no difference in TP53 expression between tumors with mutated and non-mutated TP53. In samples with mutated TP53, the median expression of CDK9 was significantly higher than in samples without mutation (FPKM 18.85 vs 20.00; *p* < 0.05) (Fig. 4b).

**Table 4:** The basic characteristics of The Cancer Genome Atlas cohort

Clinical data	Total *n* = 406 (%)
Sex	
Male	299 (73.65%)
Female	107 (26.35%)
Stage	
I	2 (0.49%)
II	129 (31.77%)
III	140 (34.48%)
IV	133 (32.76%)
Grade	
Low	20 (4.93%)
High	379 (93.35%)
Distant metastasis	
M0	193 (47.54%)
M1	11 (2.71%)
Lymph node metastasis	
N0	234 (57.64%)
N1	126 (31.03%)
Disease course	
Alive	227 (55.91%)
Dead	179 (44.09%)
TP53	
Not mutated	210 (51.72%)
Mutated	196 (48.28%)
Age (median)	69 (range 34–90)
Median follow-up time (months)	17.57 (range 0.43–168.3)

The analysis of the TCGA cohort was broadened to investigate the potential correlation with other proteins associated with p53 and CDK9 biology. However, no correlation between the expressions of CDK9, TP53, MYC, Mcl-1, CDKN1A (p21 coding gene), and CDKN2A (p16 coding gene) was statistically significant (correlation coefficients *k* < 0.2) [23–28].

## Discussion

We found that p53 is overexpressed in urothelial carcinoma tissues (Table 2, Fig. 2). P53 expression was significantly higher in tumors with distant metastasis when compared to non-metastatic tumors (*p* < 0.05). P53 has turned out to be prognostic in the NMIBC cohort, which seems to be in line with reports regarding the prognostic role of p53 in bladder cancer [29, 30].

In the early stages of BLCA, the overexpression of CDK9 and p53 seems to be a common occurrence. However, even in bladder cancers with high CDK9 expression, which seems to be a feature of less aggressive disease, high expression of p53 is associated with muscle-invasive, high-grade and metastatic cancers [22]. Those results suggest an interplay between CDK9 and p53, which may affect the progression of the disease, especially in its early stages.

### The role of p53 in urothelial carcinoma

In this study, p53 was overexpressed in urothelial carcinoma and its levels were higher in high-stage, high-grade, muscle-invasive, and metastatic disease. Those results are consistent with the recent meta-analysis published by Liao et al. and reports regarding the prognostic value of p53 in NMIBC [31, 32]. TP53 mutation is more frequent in muscle-invasive tumors when compared to non-invasive tumors (35 vs 70%), and correlates with tumor grade, stage, and disease recurrence [33–35]. The p53 loss of function often leads to the accumulation of nonfunctional p53 and manifests as overexpression in various stages of carcinogenesis [36]. Although TP53 polymorphism influences the risk of bladder cancer initiation, the overexpression of p53 is consistently associated with an increased risk of T1 NMIBC progression. Given the importance of early treatment and diagnosis, p53 overexpression may be considered an indication for more aggressive treatment [29].

Nuclear p53 phosphoprotein is a regulator of cell proliferation, cell cycle arrest, and apoptosis [37]. While its normal expression suppresses proliferation, in response to cellular stress p53 is upregulated, accumulates in the nucleus, and can initiate cell death [38]. Furthermore, wt-p53 (wild-type p53) downregulates vascular endothelial growth factor (VEGF) and basic fibroblast growth factor (bFGF) production, limiting angiogenesis [39]. Its mutation often alters related signaling pathways and could drive the initiation and progression of bladder cancer [40]. In most cases, the inactivation of the TP53 gene is caused by a sporadic loss of function mutation or negative regulation of TP53 activity. More than 75% of TP53 mutations lead to an emergence of a nonfunctional wild-type p53. It not only cannot induce cell cycle arrest, DNA repair, and apoptosis, but can also gain tumorigenic properties and drive proliferation, invasion, and survival of cells, facilitating cancer progression [37, 41, 42]. Alternatively, p53 activity can be diminished by the upregulation of its inhibitors. MDM2, an E3 ubiquitin-protein ligase, mediates the ubiquitination and degradation of p53. Therefore, deregulation of the p53/MDM2 axis may impact patients' survival, accelerate the occurrence of immune resistance and reduce the efficacy of therapy [33, 43].

### The role of CDK9 in urothelial carcinoma

The overexpression of CDK9 is frequently reported in cancers and is often associated with unfavorable prognoses. However, in some malignancies, such as PNET and neuroblastoma, its levels increase in line with cell differentiation grade [44]. In our recent study, CDK9 was overexpressed in all clinical stages of bladder cancer, while its levels decreased in line with grade and stage. Moreover, high CDK9 expression measured immunohistochemically correlated with longer patient survival. Those results were subsequently confirmed in The Cancer Genome Atlas cohort [22]. On the contrary, in Antonova et al. Study, CDK9 was upregulated in muscle-invasive bladder cancer samples when compared to non-muscle-invasive samples [45]. Rui et al. identified a novel long noncoding RNA (lncRNA) named GAS6-AS2 that contributed to the progression of bladder cancer cells through the GAS6-AS2/miR-298/CDK9 axis [46]. In this study, GAS6-AS2 knockdown in cancer cells induced G_1_ cell cycle arrest, proliferation, endothelial–mesenchymal transition and metastasis, while its overexpression correlated with worse prognosis in BLCA patients. GAS6-AS2 increased the expression of CDK9, while CDK9 knockdown antagonized the effects of GAS6-AS2 on cell migration and proliferation [46].

At first, the initiation of transcription was deemed the main checkpoint of transcriptional regulation. However, as it became apparent that RNA POL II is paused at the promoter-proximal regions of most genes in a strictly regulated manner shortly after the initiation of transcription, the control of transcription elongation gained more attention. CDK9-cyclin T1, as a key part of the PTEF-b complex required to overcome the pause and continue elongation, is now considered the central hub for transcriptional control [47, 48]. As a relatively short-lived protein, with a half-life T_1/2_ of 4–7 h, consistently expressed throughout the cell cycle, CDK9 mediates the production of anti-apoptotic proteins and enables cell division [49]. The CDK9-cyclin T1 activity seems crucial in preventing cell death in the setting of replication stress. There, the functional distinctiveness between CDK9 isoforms seems crucial. The depletion of CDK9_55_ induces double-strand DNA breaks and apoptosis, while no such activity has been reported for the CDK9_42_ isoform. CDK9_55_ interactions with Ku70, a protein partaking in the non-homologous end-joining pathway, might play a role in double-strand DNA break repair. Presumably, cyclin K, but not T is engaged in this process [15]. In addition, CDK9 forms a complex with cyclin K, which functionally substitutes for positive transcription factor b (P-TEFb) and partakes in DNA damage response as a transcriptional target for p53 [50, 51]. In the presence of DNA damage, the depletion of CDK9 and cyclin K, but not cyclin T, hinders cell cycle progression [15].

Thus, CDK9 may play a key role in preventing genome instability in the early stages of carcinogenesis. Yu D.S. et al. observed no changes in proliferation and apoptosis when CDK9 signaling was silenced in the absence of DNA damage. However, in the setting of exogenous stress, CDK9 knockdown was associated with replication fork instability and breakdown. Since only the deficit in cyclin K, but not cyclin T1 or cyclin T2, hindered the cell cycle recovery, cyclin K seems the more likely mediator of the genome-stabilizing CDK9 activity [50]. Interestingly, the role of cyclin K in DNA damage response seems ambiguous. The overexpression of cyclin K in 98G and U373MG glioblastoma cell lines and SW480 colorectal cancer cell lines suppressed cell growth after being targeted for transcription with p53 [51]. Its interplay with CDK12 seems crucial to maintaining genomic stability; the absence of cyclin K/CDK12 signaling induces spontaneous DNA damage and causes early embryonic lethality in mice [52]. On the other hand, degradation of CCNK/CDK12 in colorectal cancer inhibits cancer cell proliferation and growth in vivo [53]. Therefore, the biological effects of cyclin K activity may differ depending on the presence of exogenous DNA damage, disease stage, and the expression of its co-units.

### The prospects of p53 and CDK9 interplay

In settings of cellular stress, p53 recruits various mediators, such as cyclin K, which control the transcription of DNA damage response genes and protect cells from genomic instability [51, 52]. Cyclin T and cyclin K, forming complexes with CDK9, act independently. Therefore, the differences in signaling activity determine whether the cell will survive or undergo apoptosis [50, 54]. CDK9/cyclin T1 and p53 form a regulatory feedback loop, in which CDK9 phosphorylates the C-terminal domain of p53, activating it, while p53 binds to and activates the CDK9 promoter at the N-terminal domain [54, 55]. This mechanism seems to explain why the expression of p53 is higher in high-CDK9 tumors (Fig. 3a). Furthermore, wt-p53 might play a pivotal role in the anti-cancer activity of CDK9 inhibitors. CDK9 phosphorylates MDM2, an E3 ubiquitin-protein ligase which mediates the ubiquitination and degradation of wt-p53 [56]. The inhibition of CDK9 is capable of restoring wild-type p53 activity in tumor cells through the inhibition of MDM2 signaling [57]. However, the outcome depends on the degree of CDK9 blockade. Complete CDK9 inhibition seems to diminish the residual activity of wt-p53, while partial CDK9 blockade has the potential to restore wt-p53 function [12]. CDK9 inhibition has also been reported to limit the activity of iASPP, a preferential inhibitor of p53’s pro-apoptotic activity. In hepatocellular carcinoma cells, the overexpression of iASPP has been associated with even worse patients’ overall survival than MDM2 overexpression [11]. Given that CDK9 is involved in the regulation of two main p53 inhibitors, its blockade may lead to the restoration of wild-type p53 functions, which has been reported to suppress tumor growth in tumors with a low frequency of p53 mutations. Therefore, CDK9 inhibitors might be most effective in lower-grade bladder cancers, where p53 mutations are still rarer and the genome is more stable than in high-grade tumors [11, 12, 58].

## Conclusion

P53 is overexpressed in bladder cancer and its high expression correlates with the occurrence of metastasis. In non-muscle-invasive bladder cancer, p53 is a predictor of shorter overall survival, and shorter progression-free survival, while its expression increases in line with cancer grade. CDK9 is overexpressed in bladder cancer and correlates with favorable clinical features and longer patient survival. Although we found no correlations between the expression of p53 and CDK9, the levels of p53 were higher in cancers with high CDK9 expression. In high-CDK9 cancers, p53 was associated with high-grade and muscle-invasive cancers. Since the inhibition of CDK9 in other malignancies was reported to downregulate the expression of two main p53 inhibitors, MDM2 and iASPP, then its concurrent blockade may be an interesting approach to reactivate wild-p53 activity. Nevertheless, to this day, no clinical trials regarding the use of CDK9 inhibitors in bladder cancer have been conducted.

## Data Availability

The results from the CDK9 group have been published in our recent article and are available on request from the corresponding author; reference [16]. The data presented in this study are available on request from the corresponding author. The data are not publicly available due to ethical restrictions. This study includes publicly available data from The Cancer Genome Atlas database; references: [23–28].
